# Reliability, validity and administrative burden of the community reintegration of injured service members computer adaptive test (CRIS-CAT)”

**DOI:** 10.1186/1471-2288-12-145

**Published:** 2012-09-17

**Authors:** Linda Resnik, Matthew Borgia, Pensheng Ni, Paul A Pirraglia, Alan Jette

**Affiliations:** 1 Providence VA Medical Center, 830 Chalkstone Avenue, Providence, RI, 02908, USA; 2 Department of Health Services, Policy and Practice, Brown University, 121 South Main Street, Providence, RI, 02903, USA; 3 Health & Disability Research Institute, Boston University School of Public Health, 580 Harrison Avenue, Boston, MA, 02118, USA; 4 Department of Health Policy and Management, Health & Disability Research Institute, Boston University School of Public Health, 580 Harrison Avenue, Boston, MA, 02118, USA

## Abstract

**Background:**

The Computer Adaptive Test version of the Community Reintegration of Injured Service Members measure (CRIS-CAT) consists of three scales measuring Extent of, Perceived Limitations in, and Satisfaction with community integration. The CRIS-CAT was developed using item response theory methods. The purposes of this study were to assess the reliability, concurrent, known group and predictive validity and respondent burden of the CRIS-CAT.

The CRIS-CAT was developed using item response theory methods. The purposes of this study were to assess the reliability, concurrent, known group and predictive validity and respondent burden of the CRIS-CAT.

**Methods:**

This was a three-part study that included a 1) a cross-sectional field study of 517 homeless, employed, and Operation Enduring Freedom / Operation Iraqi Freedom (OEF/OIF) Veterans; who completed all items in the CRIS item set, 2) a cohort study with one year follow-up study of 135 OEF/OIF Veterans, and 3) a 50-person study of CRIS-CAT administration. Conditional reliability of simulated CAT scores was calculated from the field study data, and concurrent validity and known group validity were examined using Pearson product correlations and ANOVAs. Data from the cohort were used to examine the ability of the CRIS-CAT to predict key one year outcomes. Data from the CRIS-CAT administration study were used to calculate ICC (2,1) minimum detectable change (MDC), and average number of items used during CAT administration.

**Results:**

Reliability scores for all scales were above 0.75, but decreased at both ends of the score continuum. CRIS-CAT scores were correlated with concurrent validity indicators and differed significantly between the three Veteran groups (P < .001). The odds of having any Emergency Room visits were reduced for Veterans with better CRIS-CAT scores (Extent, Perceived Satisfaction respectively: OR = 0.94, 0.93, 0.95; P < .05). CRIS-CAT scores were predictive of SF-12 physical and mental health related quality of life scores at the 1 year follow-up. Scales had ICCs >0.9. MDCs were 5.9, 6.2, and 3.6, respectively for Extent, Perceived and Satisfaction subscales. Number of items (mn, SD) administered at Visit 1 were 14.6 (3.8) 10.9 (2.7) and 10.4 (1.7) respectively for Extent, Perceived and Satisfaction subscales.

**Conclusion:**

The CRIS-CAT demonstrated sound measurement properties including reliability, construct, known group and predictive validity, and it was administered with minimal respondent burden. These findings support the use of this measure in assessing community reintegration.

## Background

Over 2 million U.S. troops have been involved in the conflicts in Iraq and Afghanistan (Operation Enduring Freedom / Operation Iraqi Freedom) [OEF/OIF] since 2001. The mortality and combat casualty rates from these conflicts are regularly reported [1]. As of April 19, 2011 there have been 5955 deaths and 42,963 wounded in OEF/OIF combined [1]. However, the long term morbidity and associated social and economic impact of these wars impact far more service members than previous conflicts. In fact, experts estimate that more than 790,000 OEF/OIF veterans have service-related health problems which will lead them to seek disability benefits [2].

Some OEF/OIF Veteran casualties survive due to a combination of improved body armor and advancements in battlefield care and are seeking disability benefits for these severe and complex physical wounds. Improvements in body armor protect the torso, but not the brain or extremities, which has resulted in many cases with multiple and severe injuries [3,4], as well as traumatic brain injury (TBI). TBI has been identified as the “signature injury” of these wars, and has been reported in almost 1/3 of those injured in combat [5,6]. Blast injury is the cause of the majority of cases of TBI among OEF/OIF Veterans. Consequences of blast-related mild TBI are pervasive, as TBI can result in impairments in decision-making associated with patterns of impulsive [7] and/or aggressive choices [8,9].

The vast majority of injured service members who have been deployed to these wars have sustained less serious physical injuries; and many more have “invisible” wounds of war, affecting them psychologically and emotionally. Hoge estimated that 19.1% of OIF Veterans and 8.5% of OEF Veterans reported a mental health problem [10]. A higher prevalence of mental health conditions was reported for those Service members who sought care from the VA, with over 35% of those OEF/OIF Veterans receiving diagnosis for a mental health condition, the most prevalent of which were PTSD (21.8%) and depression (17.4%) [11].

Returning service members may struggle with an array of problems in reintegration into their community, which include problems related to psychological health, marital and financial difficulties, alcohol or substance abuse, and car and motorcycle accidents. A recent study of OEF/OIF Veterans accessing VA health services found approximately half had problems with community reintegration, such as problems controlling anger (52%), diminished participation in community activities (49%), difficulty getting along with an intimate partner (42%), problems in employment (20%) and legal problems (20%) [12]. Recent data from the Army show an overall increase in the number of divorces since the start of OEF and OIF, especially in female soldiers [2]. A recent study of OEF/OIF/Operation New Dawn (OND) veterans referred for behavioral health evaluation found that more than 75% of patients with partners reported some family readjustment issues, with 66% reporting this occurring on a weekly basis [12].

One of the key roles of the Department of Veterans Affairs (VA) is to help injured Veterans return to full participation in community life roles. Given this mission, a tool to measure community reintegration is critically needed to track Veteran functioning in this domain, and to assess the impact of VA treatment and policy. At this time, there is no widely accepted measure that serves as the “gold standard” of Veteran’s community reintegration [13].

The Community Re-integration of Service Members (CRIS) and a computer adaptive test version of the measure, the CRIS-CAT, were developed to address this gap [14]. These instruments aim to measure the latent trait of community reintegration. Latent traits are those that are not directly observable. However, prior to widespread adoption, studies are needed to determine test-retest reliability, concurrent, known group and predictive validity. This type of information is necessary for monitoring of Veteran functioning and for targeting treatment to Veterans at risk for adverse outcomes.

Thus, the purposes of this companion study were to 1) assess the reliability and concurrent, known group, and predictive validity of the newly developed CRIS-CAT scales and 2) assess respondent burden. We hypothesized that lower CRIS-CAT scores at Visit 1 would be associated with negative outcomes one year later including: worsening of employment status, less housing stability, change in quality of life (measured by SF-12 scores) and more ER visits.

## Methods

### Overview

**Figure 1:**
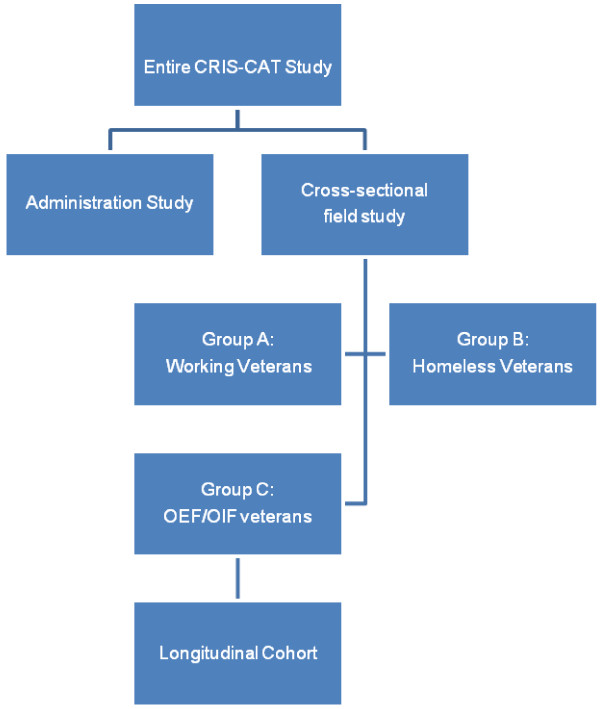
Overall organization of the study.

The 3-part study involved 1) a cross-sectional field study, 2) a one year follow-up study of OEF/OIF Veterans from the field study (cohort study), and 3) a separate 50 person study of CRIS-CAT administration. The overall organization of the study is shown in Figure 1. The methods for each sub-study are described below. All sub-studies were approved by the Institutional Review Board of the Providence VA Medical Center. All subjects provided informed consent.

### Field study

#### Sample

The field study included a convenience sample of 517 Veterans. Subjects were English-speaking men and women, ages 18 and older, recruited from 3 sub-groups (that we expected to represent a wide spectrum of community integration). Subjects recruited into group A were non-OEF/OIF Veterans under 60 years old, observed to have good community integration, i.e. those with housing stability and steady employment and who screen negative for depression, PTSD, and substance abuse. Subjects for group B were either considered homeless (or insecurely housed) and/or chronically unemployed non-OEF/OIF Veterans under 60 years of age who were expected to have lower community integration. Subjects were recruited through the PVAMC primary care and PTSD services, community and military reserve sites in Rhode Island, Southeastern Massachusetts and nearby Connecticut, and homeless shelters in the region. Subjects for group C were any Veterans who had been deployed in support of OEF/OIF.

#### Data collection

Subjects participated in a single session of data collection in which trained interviewers administered the entire CRIS item set ( Additional file 1. Appendix A) and asked questions about demographics and health history. Interviewers read the standardized instructions and questions from a script, and showed the subject the choice of response categories where appropriate. Subjects’ responses were recorded by the interviewer on study laptops.

## Measures

### The CRIS-CAT

The CRIS assesses community reintegration through the assessment of participation in life roles as defined by the International Classification of Functioning, Disability and Health (ICF) [15], an approach consistent with recent recommendations from the VA’s State of the Science Working Group on Measurement of Community Reintegration [16]. Items on the CRIS cover 9 aspects of participation: (1) Learning and Applying Knowledge, (2) General Tasks and Demands, (3) Communication, (4) Mobility, (5) Self-care, (6) Domestic Life, (7) Interpersonal Relationships, (8) Major Life Areas, and (9) Community, Social and Civic Life. The CRIS’s scales measure these aspects in three dimensions. The Extent of Participation scale asks the respondent to indicate how often he or she experiences or participates in specific activities. The Perceived Limitations in Participation scale asks the respondent to indicate his or her perceived limitations in participation. Lastly, the Satisfaction with Participation scale asks the respondent to indicate the degree of satisfaction with participation.

Computer Adaptive Test (CAT) methodologies use a computer interface to select and administer items tailored to the unique level of the respondent [17]. Use of CAT based measures reduces respondent burden by allowing administration of fewer items as compared to fixed form measures. CAT applications require a set of items in functional domains that consistently scale along a dimension of low to high functional proficiency in the domain of interest. In previous work we developed the CRIS-CAT by drawing upon a large item pool: Extent of Participation (77 item pool), Perceived Limitation in Participation (144 item pool) and Satisfaction with Participation (86 item pool) [16].

We employed Item Response Theory methods to develop a CAT version of the CRIS that allows for accurate, precise, and reliable measurement of community reintegration with reduced respondent burden. Our IRT models showed that the CRIS-CAT scales were unidimensional. CRIS-CAT scores were estimated from subjects’ responses to the full item pool based on the Weighted Maximum Likelihood Estimation Method [18]. The CAT stopping rules were set at a minimum of 10 items and a maximum of 20 items and a SE of the latent trait set to 0.32. Reliability of the CAT under these conditions was equal to 0.9. Our data simulations showed that the CRIS-CAT scales had good fit, and had excellent precision as compared to the full item set [16]. These findings suggested that the CRIS-CAT was a sound measure of community reintegration with reduced respondent burden.

### Additional measures

We collected data on measures which we believed would be correlated with CRIS-CAT scores if the instruments were measuring community integration. We expected to observe similar relationships to those that we found in our earlier study of the CRIS fixed form measure [14].These measures included two scales from the Craig Handicapped Assessment and Reporting Technique (CHART) [[19], the SF-36 V, [20] and the Quality of Life (QOL) measure [21]. The CHART social integration subscale consists of 6 questions about extent of participation in and maintenance of customary social relationships [22]. The CHART occupation subscale consists of 7 questions about extent of participation in occupational activities customary to a person's sex, age, and culture [22]. The SF-36 V, a version of the SF-36 is adapted for the Veteran population [20]. Using SF-36 V responses we calculated the physical component summary score (PCS) and the mental component summary score (MCS) as well as the Physical Functioning Scale (PF-10) [23]. The Quality of Life scale (QOL) consists of 16 questions that assess satisfaction with independent living and self-care activities [21].

Other demographic information that was collected at Visit 1 included age, gender, marital status, race, ethnicity, education, income, employment, parenthood, presence of children in the home, and housing type. Marital & Relationship Status was assessed using a question from the U.S. Census Current Population Survey (CPS) which classified marital status as now married, widowed, divorced, separated, and never married. We re-categorized this variable into three groups: married, never married and other (widowed, divorced or separated). Race was categorized as Caucasian, Black, Mixed and Other. Ethnicity was categorized as Hispanic or non-Hispanic. Education was categorized as less than high school, high school diploma, GED, some college, college graduate and postgraduate education. Income was assessed using a question from the Behavioral Risk Factor Surveillance System (BRFSS) to estimate total household income in the previous year. Income was classified into three categories: less than $25 K, between $25 K and $50 K, and over $50 K. Employment was assessed by 4 questions drawn from the U.S. Census Current Population Survey (CPS) which ask if the respondent was working, a student, or participating in regular volunteer activities. Employment was classified as unemployed, not working due to disability or on medical hold, working part-time or training, and working full-time. Parenthood was defined as having children or stepchildren yes or no. Housing Type was determined by asking respondents to indicate their type of residence (outdoors, staying with friend, vet home, house, apartment, or other).

Additionally, we collected data on OEF/OIF Veteran status, whether or not the Veteran had ever been deployed, and if so how many times, and the number of months since returning from deployment. We also asked subjects to indicate whether they had ever been diagnosed with depression, alcohol/drug abuse, Post Traumatic Stress Disorder (PTSD), or other mental illness.

### Cohort study

#### Sample

The sample for the cohort study consisted of Veterans from Group C (OEF/OIF) who had Visit 1 interviews between the dates of February 1, 2008 and February 28, 2010.

#### Data collection

Subjects in the cohort study participated in two visits. The first visit occurred as part of the field study and a second visit took place one year later (Visit 2).

## Measures

We collected all measures used in the field study (described above). In addition, we collected data on the following outcomes one year after Visit 1, emergency room (ER) use within the prior year, housing stability, change in employment status, change in marital status, HRQL, and new diagnosis of mental health condition. We hypothesized, based upon the literature, that these key 1 year outcomes might be associated with community reintegration scores.

ER use in the prior year was collected using two methods: self-report by the Veteran and abstraction of the VA medical records using the Austin Automated Database. Housing Stability was determined by asking respondents to indicate the number of times that they had moved in the past year (number of moves). For data analysis this variable was categorized into three classes: no moves, one move, and two or more moves.

Change in Employment Status was categorized into three groups: improved, worse, and no change. Improved Employment Status was defined as change from Visit 1 to Visit 2 from not working to working, i.e. unemployed or not working due to medical hold at baseline to working full or part-time at 1 year follow-up. Worse employment was defined as change from working to not working, i.e. working full or part time at baseline to being unemployed or not working due to medical hold at 1 year follow-up. We classified subjects as having no change in employment if their overall employment status (working or not working or retired) stayed the same between visits.

Change in Marital Status at Visit 2 was categorized into three groups: newly married, no longer married and unchanged. Newly married status was defined as change from any non-married group at baseline to married at 1 year follow-up. The no longer married status was defined as change from married at baseline to any non-married group at 1 year follow-up. We classified any subjects who remained married or remained in the non-married groups as having an unchanged marital status.

Health Related Quality of Life (HRQL) was assessed using the 12 question SF-12 (embedded in the SF-36 items at Visit 1, and asked by themselves at the follow-up visit) which evaluated two global health constructs: the physical component summary (PCS) and the mental component summary (MCS) [24].

New Diagnosis of Mental Illness was determining using mental health diagnoses codes abstracted from the subject’s medical records at two time points (Visit 1 and Visit 2). We categorized the ICD-9 codes into 21 diagnosis groups as follows: Presence of Adjustment Disorders, Affective Disorders, Alcohol Dependence Syndrome, Anxiety Disorders, Attention Deficit, Drug Dependence, Drug Psychoses, Eating Disorders, Gender Identity Disorders, Impulsive-Control Disorders, Mild Mental Retardation, Mood Disorders, Neurotic Disorders, Non-Dependent Abuse of Drugs, Personality Disorders, Post-concussion Syndrome, Psychotic Disorders, PTSD, Sexual Dysfunctions, Somatoform Disorders, and Unspecified Disorders. These diagnostic groups were based upon the classification system as defined by the Diagnostic Statistical Manual-IV (DSM-IV) [25]. We then modified this classification system so as to include categories comprised of diagnoses that we expected to be prevalent and/or important in this population (e.g. PTSD, alcohol abuse or dependence, substance use or dependence). If any of these disorders were present at Visit 2 but not present at Visit 1 we considered the subject to have a new mental health condition.

### Administration study

#### Sample

The sample for the administration study was a convenience sample of 50 Veterans (ages 18–59) from the PVAMC who had not participated in the earlier studies.

#### Data collection

Subjects in the CRIS-CAT Administration Study participated in two data collection visits conducted within 1 week. Subjects completed the CRIS-CAT at both visits using newly develop CAT software. Demographic data were collected at the first visit. In order to assess respondent burden we tracked number of items administered in each scale within the software database.

### Statistical analyses

Data from the field study were used to examine reliability of the CAT, and test concurrent and known groups validity. We examined descriptive characteristics of field study participants, and compared characteristics for each subsample.

Reliability represents the degree to which the differences across subject scores are due to real differences in scale (true variance) as opposed to measurement error. By assuming the latent trait under the partial credit model has a standard normal distribution, the conditional reliability of the CRIS-CAT scales was examined as 1/(1 + (standard error)^2 ), which was a function of latent trait level. The standard error of the person score estimates was derived from the information function. Any section of the reliability function that was greater than 0.70 served to indicate adequate reliability [26].

Concurrent validity of the CRIS-CAT was examined by exploring Pearson product correlations of the CRIS-CAT scales with existing measures that assess specific community reintegration dimensions. We expected to find similar correlations to those reported in our earlier research on the CRIS fixed form measure. (i.e. correlations between the CRIS fixed form measure and the 36-Item Short Form Health Survey scales of role physical, role emotional and social functioning of 0.25-0.80 [13,14] and a correlation between the CRIS scores and QOL of 0.57-0.79.) [13].

We performed ANOVAs to examine whether the CRIS-CAT scores differed in Veterans from the 3 groups as expected: the homeless group, the working group and the OEF/OIF group.

#### Statistical methods - cohort

Data from the cohort study were used in the examination of the CRIS-CAT’s predictive validity. We examined the CRIS-CAT measure’s ability to predict key one year outcomes including change in marital status, employment status, housing stability, self-reported ER visit frequency, frequency of VA ER use, SF-12 scores, and new diagnoses of mental health condition. Likelihood of change in marital status was modeled using the three-level category of change in marital status. Similarly, likelihood of change in employment status between Visit 1 and Visit 2 was modeled using the three-level category of change in employment. We ran 3 separate multinomial regression models to predict change in employment status using three CRIS-CAT subscale scores at baseline as independent variables. Similarly, we ran 3 separate multinomial regression models to predict likelihood of housing stability.

We developed three separate logistic regression models to predict the likelihood of any self-reported ER visits for participants in the cohort. We also examined 1 year ER use for all field study participants as documented in the VA medical record. We ran three separate logistic regression models to predict the likelihood of any recorded ER visits based on the three CRIS subscale scores, using data abstracted from the Austin Automated Database. For the cohort participants, we ran three separate logistic regression models to predict the likelihood of the diagnosis of any new mental health condition based on the three CRIS-CAT subscale scores using the abstracted medical data. CRIS-CAT scores were used to predict SF-12 scores one year later while controlling for Visit 1 SF-12 scores in the linear regressions.

#### Statistical methods - administration study

Data from the 50-person CRIS-CAT administration study were used to assess respondent burden, test-retest reliability, estimate the minimal detectable change. We calculated CRIS-CAT scores for each visit and examined the number of items used by the CAT to estimate scores. Reliability of the 3 CRIS-CAT scales was evaluated using Shrout & Fleiss intraclass correlation coefficients which were calculated using SPSS (PASW Statistics 18). ICC(2,1) is a two-way mixed effects single measure of reliability, where the target is a random effect, the number of measurements on each target is a fixed effect, and the unit of analysis is the individual measurement instead of the mean of measurements [27].

Using a classical test theory approach, ICCs were then used to calculate the Standard Error of the Measurement (SEM) and Minimum Detectable Change (MDC). The SEM is the standard error in an observed score related to measuring with a particular test that obscures the true score. It is estimated by the standard deviation of the instrument multiplied by the square root of one minus its reliability coefficient [28]. In statistical terms, the MDC, also called smallest detectable change or smallest real change shows which changes fall outside the measurement error of the health status measurement (either based on internal or test-retest reliability in stable persons) [29,30]. The Standard Error of the Measurement (SEM) and MDC were calculated at 95% confidence and 90% confidence levels [31].

To assess potential bias due to loss to follow-up we examined characteristics of participants in the cohort study and compared characteristics of eligible subjects lost to follow-up and those with complete data at one year.

## Results

### Field study

Characteristics of the field study sample are shown in Table 1. The sample consisted of 517 Veterans; 69 in Group A (those with a high degree of community reintegration), 99 in Group B (homeless Veterans) and 332 in Group C (OEF/OIF Veterans). There were 17 additional Veterans who were screened into one of the above groups, but who, on inspection of their data, were excluded since they did not meet inclusion criteria for the study.

**Table 1:** Descriptive characteristics of subjects in the field study by group

	**Group A (N = 69)**	**Group B (N = 99)**	**Group C (N = 332)**	**Other (N = 17)**	**ALL (N = 517)**
	**Mean (SD) range**	**Mean (SD) range**	**Mean (SD) range**	**Mean (SD) range**	**Mean (SD) range**
**Age**	47.7 (8.7) 25-60	51.1 (7.0) 31-60	34.5 (9.9) 19-59	43.8 (8.4) 34-58	39.7 (11.7) 19-60
	**Frequency (%)**	**Frequency (%)**	**Frequency (%)**	**Frequency (%)**	**Frequency (%)**
**Gender**					
Male	49 (71.0)	83 (83.8)	285 (85.8)	16 (94.1)	433 (83.8)
Female	20 (29.0)	16 (16.2)	47 (14.2)	1 (5.9)	84 (16.3)
**Race**					
White	50 (72.5)	70 (71.4)	262 (79.2)	12 (70.6)	394 (76.5)
Black	8 (11.6)	17 (17.4)	16 (4.8)	3 (17.7)	44 (8.5)
Other	9 (13.0)	3 (3.1)	31 (9.4)	1 (5.9)	44 (8.5)
Mixed	2 (2.9)	8 (8.2)	22 (6.7)	1 (5.9)	33 (6.4)
**Hispanic**	5 (7.3)	5 (5.1)	32 (9.7)	1 (5.9)	43 (8.4)
**Has Children**	53 (76.8)	73 (73.7)	179 (53.9)	12 (70.6)	317 (61.3)
**Education**					
Less than High School	1 (1.5)	6 (6.1)	0 (0.0)	0 (0.0)	7 (1.4)
High School	11 (15.9)	32 (32.3)	73 (21.9)	5 (29.4)	121 (23.4)
GED	1 (1.5)	16 (16.2)	12 (3.6)	1 (5.9)	30 (5.8)
Some college	30 (43.5)	34 (34.3)	152 (45.8)	5 (29.4)	221 (42.8)
College	16 (23.2)	8 (8.1)	70 (21.1)	5 (29.4)	99 (19.2)
Post Grad	10 (14.5)	3 (3.0)	25 (7.5)	1 (5.9)	39 (7.5)
**Employment status**					
Unemployed	0 (0.0)	29 (29.3)	67 (20.2)	3 (17.7)	99 (19.2)
Not working due to disability/medical hold	0 (0.0)	61 (61.6)	23 (7.0)	3 (17.7)	87 (16.9)
Working part-time/training	9 (13.0)	8 (8.1)	38 (11.5)	1 (5.9)	56 (10.9)
Working full-time	59 (85.5)	1 (1.0)	200 (60.6)	10 (58.8)	270 (52.4)
Retired	1 (1.5)	0 (0.0)	2 (0.6)	0 (0.0)	3 (0.6)
**Income**					
Less than $25 K	10 (14.5)	75 (75.8)	86 (26.1)	2 (11.8)	173 (33.7)
$25 k to $50 k	20 (29.0)	17 (17.2)	101 (30.7)	8 (47.1)	146 (28.4)
Over $50 k	39 (56.5)	7 (7.1)	142 (43.2)	7 (41.2)	195 (37.9)
**Marital Status**					
Unmarried	19 (27.5)	26 (26.3)	130 (39.2)	6 (35.3)	181 (35.0)
Married	37 (53.6)	17 (17.2)	151 (45.5)	7 (41.2)	212 (41.0)
Divorced, Separated or Widowed	13 (18.9)	56 (56.5)	51 (15.3)	4 (23.5)	124 (24.0)
**Residence**					
Outside	0 (0.0)	2 (2.0)	0 (0.0)	0 (0.0)	2 (0.4)
Staying with friend	0 (0.0)	11 (11.1)	35 (10.5)	1 (5.9)	47 (9.1)
Vet Home	0 (0.0)	28 (28.3)	3 (0.9)	0 (0.0)	31 (6.0)
House	19 (27.5)	31 (31.3)	88 (26.5)	6 (35.3)	144 (27.9)
Apartment	50 (72.5)	22 (22.2)	190 (57.2)	10 (58.8)	272 (52.6)
Other	0 (0.0)	5 (5.1)	16 (4.8)	0 (0.0)	21 (4.1)
**Depression Diagnosis**	0 (0.0)	70 (70.7)	83 (25.7)	1 (5.9)	154 (30.3)
**PTSD Diagnosis**	0 (0.0)	51 (53.1)	90 (27.7)	3 (17.7)	144 (28.4)
**Mental Illness Diagnosis**	1 (1.5)	48 (50.0)	56 (17.0)	0 (0.0)	105 (20.6)
**Alcohol/Drug abuse Diagnosis**	0 (0.0)	68 (68.7)	67 (20.2)	6 (35.3)	141 (27.3)

**Figure 2:**
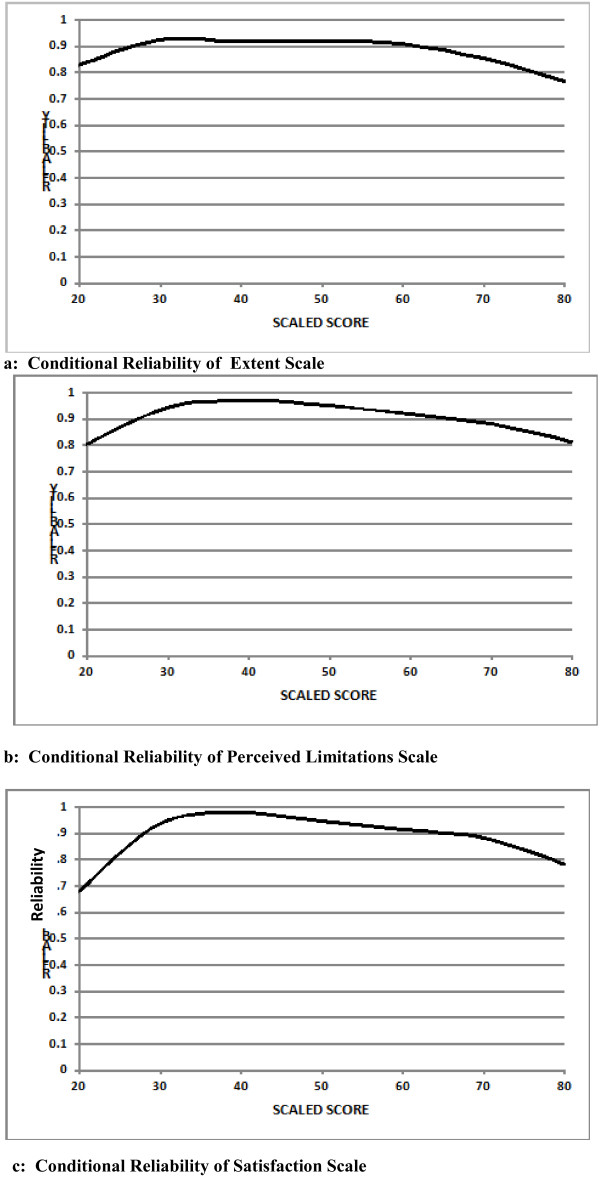
**a Conditional reliability of extent scale. b** Conditional reliability of perceived limitations scale.** c**: Conditional reliability of satisfaction scale.

Figures 2a, b, and c show the level of scale score conditional reliability [32] across each CRIS-CAT scale continuum. Reliability was above 0.75 for most of the scale range, but decreased at both ends of the continuum where there were fewer items available for administration.

**Figure 3:**
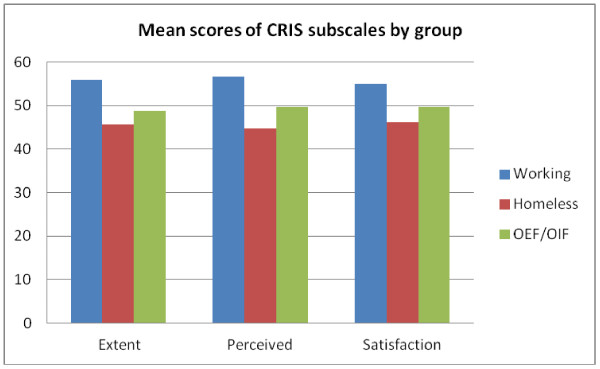
CRIS subscale scores at Visit 1.

Results of the concurrent validity analyses are shown in Table 2. All CRIS-CAT scales were strongly correlated with SF-36 Role Physical, Social Functioning, Role Emotional and Physical Functioning, and with QOL, negatively correlated with the number of difficulties in Activities of Daily Living (ADL) and moderately correlated with the CHART subscales. Known group validity analyses conducted via ANOVAs determined that CAT subscale scores differed significantly between the three sample groups (P < .000 for all comparisons) (Figure 3). Veterans in the homeless group had the lowest CRIS-CAT scores (Extent mn 45.6 ±8.9), (Perceived mn 44.7 ±7.3) and (Satisfaction mn 45.1 0 ± 7.2) and subjects in the working group had the highest scores (Extent mn 55.8 ±8.2), (Perceived mn 56.6 ±11.5) and (Satisfaction mn 54.9 ±11.0). OEF/OIF Veterans had average scores between the other two groups (Extent mn 48.7 ±9.2), (Perceived mn 49.6 ±8.6) and (Satisfaction mn 49.6 ± 9.0).

**Table 2:** Concurrent and discriminant validity of CRIS-CAT scales: Pearson product correlations data from the field study N = 500 (all p values <0.0001)

**Measure**	**Extent of participation**	**Perceived limitations**	**Participation satisfaction**
	***R***	***R***	***R***
Quality of Life Scale	0.71	0.69	0.76
Activities of Daily Living	−0.38	−0.36	−0.34
Occupation (CHART)	0.32	0.31	0.27
Social Integration (CHART)	0.34	0.28	0.36
Physical Function (SF-36)	0.49	0.38	0.37
Role Physical (SF-36)	0.48	0.45	0.44
Role Emotional (SF-36)	0.63	0.60	0.55
Social Functional (SF-36)	0.66	0.66	0.58

### Cohort study

**Figure 4:**
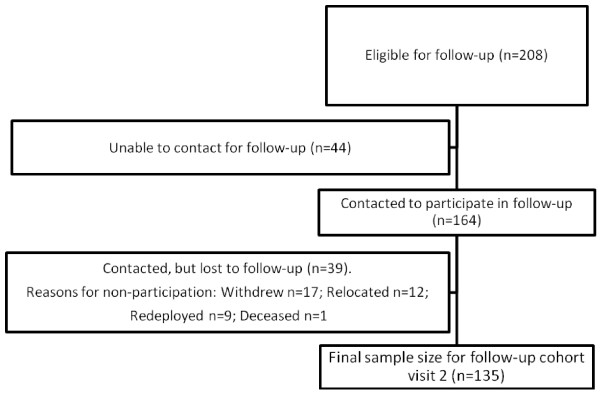
Flow of participants in the study.

One hundred and thirty five of the 208 OEF/OIF Veterans from Visit 1 returned for Visit 2. Figure 4 shows the number of subjects who were not reached for follow-up, as well as those who were reached and the reasons that they declined to participate. Table 3 shows the characteristics of those subjects from the cohort who were lost to follow-up and those who completed the second visit. Compared to subjects who completed Visit 2, lost to follow-up subjects were significantly younger (p < 0.001), had been deployed more recently (p < 0.05), had lower incomes (p < 0.01), were less likely to have children (p < 0.05), be married (p < 0.05), or have an apartment or house (p < .01), and had fewer instances of depression (p < 0.05) and alcohol/drug abuse diagnoses (p < 0.05). One hundred twenty two of the subjects included in the cohort had data available from the VA medical record.

**Table 3:** **Characteristics of participants in the longitudinal cohort study and administration study s**: P-values below 0.05 (*), 0.01 (**) and 0.001 (***)

	**Candidates for cohort study**			**Administration study**
	**Lost to follow-up (N = 73)**	**Followed at 1 year (N = 135)**	**P**	
	**Mean (SD) Range**	**Mean (SD) Range**		**Mean (SD) Range**
**Age**	30.6 (8.8) 20-55	36.7 (10.0) 21-59	**0.000**	48.2 (9.8) 24-59
**Month since return from deployment**	22.1 (19.9) 0-68	28.5 (19.0) 1-96	**0.015**	
**SF-12 MCS**	45.1 (9.2) 43.0-47.3	43.7 (11.9) 41.6-45.7	0.1863	
SF-12 PCS	41.7 (5.2) 40.4-42.9	41.2 (6.0) 40.1-42.2	0.2727	
CRIS-CAT Extent	49.7 (10.1) 47.3-52.0	47.6 (9.0) 46.0-49.1	0.0638	
CRIS-CAT Perceived	50.1 (9.7) 47.8-52.4	48.9 (7.6) 47.6-50.2	0.1650	
CRIS-CAT Satisfaction	49.7 (9.3) 47.5-51.9	49.1 (8.4) 47.7-50.6	0.3361	
	**Frequency (%)**	**Frequency (%)**		**Frequency (%)**
**Gender**			*0.196*	
Male	57 (78.1)	115 (85.2)		35 (70.0)
Female	16 (21.9)	20 (14.8)		15 (30.0)
**Race**			0.266	
White	52 (71.2)	111 (82.2)		34 (69.4)
Black	4 (5.5)	6 (4.4)		3 (6.1)
Other	11 (15.1)	10 (7.4)		3 (6.1)
Mixed	6 (8.2)	8 (5.9)		9 (18.4)
**Ethnicity**	12 (16.4)	11 (8.2)	0.072	3 (6.1)
**Has Children**	33 (45.2)	82 (60.7)	**0.031**	50 (100.0)
**Education**			0.441	
Less than High School	0 (0.0)	0 (0.0)		1 (4.0)
High School	21 (28.8)	25 (18.5)		8 (16.0)
GED	3 (4.1)	6 (4.4)		1 (2.0)
Some college	34 (46.6)	64 (47.4)		23 (46.0)
College	11 (15.1)	29 (21.5)		12 (24.0)
Post Grad	4 (5.5)	11 (8.2)		4 (8.0)
**Employment status**			0.058	
Unemployed	21 (28.8)	20 (14.8)		5 (10.0)
Not working due to disability/medical hold	3 (4.1)	14 (10.4)		15 (30.0)
Working part-time/training	10 (13.7)	18 (13.3)		5 (10.0)
Working full-time	39 (53.4)	83 (61.5)		19 (38.0)
Retired	0 (0.0)	0 (0.0)		6 (12.0)
**Income**			**0.013**	
Less than $25 K	26 (35.6)	27 (20.0)		16 (32.0)
$25 k to $50 k	25 (34.3)	42 (31.1)		
Over $50 k	22 (30.1)	66 (48.9)		21 (42.0)
**Marital Status**			**0.014**	
Unmarried	37 (50.7)	44 (32.6)		10 (20.0)
Married	22 (30.2)	68 (50.4)		25 (50.0)
Divorced, Separated or Widowed	14 (19.2)	23 (17.1)		15 (30.0)
**Residence**			**0.002**	
Outside	0 (0.0)	0 (0.0)		0 (0.0)
Staying with friend	12 (16.4)	8 (5.9)		1 (2.0)
Vet Home	0 (0.0)	2 (1.5)		2 (4.0)
House	18 (24.7)	37 (27.4)		17 (34.0)
Apartment	34 (46.6)	85 (63.0)		29 (58.0)
Other	9 (12.3)	3 (2.2)		1 (2.0)
**Depression Diagnosis**	14 (19.7)	45 (33.8)	**0.034**	20 (40.8)
**PTSD Diagnosis**	16 (22.5)	44 (33.6)	0.101	20 (40.0)
**Mental Illness Diagnosis**	8 (11.3)	23 (17.0)	0.271	13 (26.0)
**Alcohol/Drug abuse Diagnosis**	8 (11.1)	32 (23.7)	**0.029**	17 (34.0)
**New Diagnosis of Mental Illness**	0 (0.0)	35 (28.7)	**0.000**	N/A
**Imputed MCS score at Visit 2**	45.0	44.8		
**Imputed PCS score at Visit 2**	50.4	46.3		

**Figure 5:**
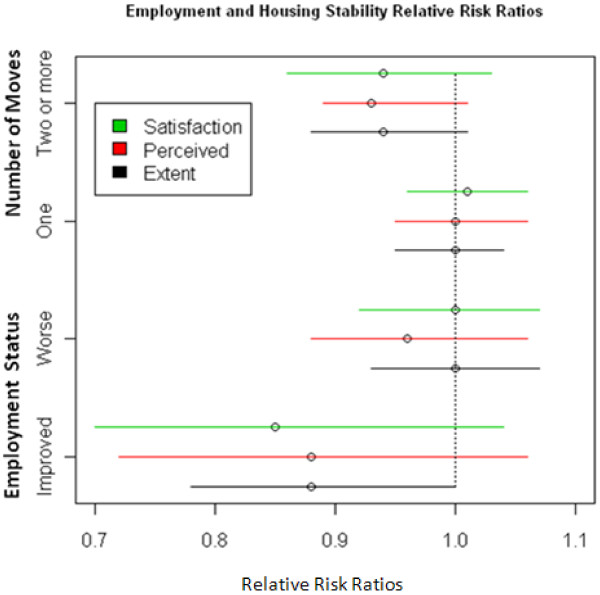
Results of separate multinomial logistic regression predicting change in employment status, and housing stability for Persons in the Longitudinal Cohort Study.

**Figure 6:**
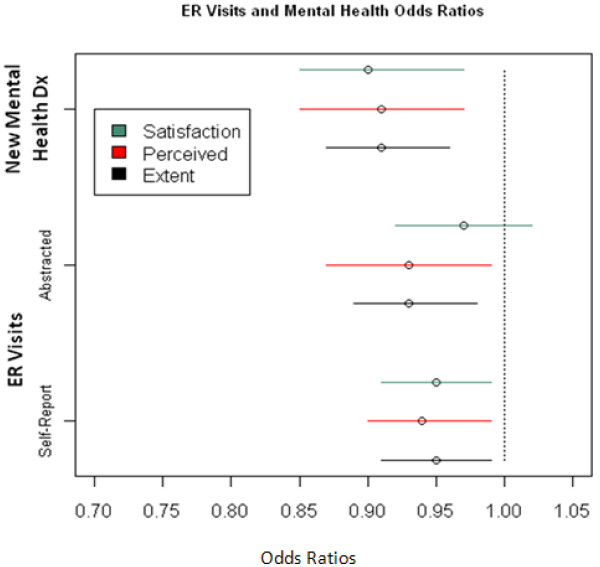
Results of separate logistic regression models predicting any self reported or abstracted ER visit use, new diagnosis of mental health disorder one year after Visit 1.

Only 3.7% of the cohort group reported a change in their marital status between Visit 1 and one year follow-up. Whereas 11.5% reported a change in employment status (8.4% for the worse) (Table 4). Although the majority of subjects had not moved in the prior year, 22.2% had moved once, and 8.9% had moved more than once. Contrary to our hypotheses, CRIS-CAT scores at Visit 1 were not predictive of change in marital status (not shown) or change in employment status or housing stability one year later (Figure 5). The odds of having any self-reported or abstracted ER visits were reduced for those with higher CRIS scores; Extent, Perceived, Satisfaction subscales respectively (OR 0.94, 0.93, 0.95, P < .05). (Figure 6) The odds of having any abstracted ER visits were reduced for those with higher CRIS Extent or Perceived scores respectively (OR 0.93, 0.93, .097; P < .05). (Figure 6) The odds of having a new diagnosis of a mental health condition one year later were lower for persons with higher CRIS-CAT scores at Visit 1 Extent, Perceived, Satisfaction subscales respectively (OR 0.91, 0.91, 0.90; P < 001) (Figure 6). CRIS-CAT scores at Visit 1 were predictive of SF-12 PCS and MCS scores 1 year later. (Table 5)

**Table 4:** Key outcomes at V2: longitudinal cohort study

	**One year (N = 135)**
	**Mean (SD) Range**
**Change in PCS scores**	5.1 (12.3) -26.3, 30.6
**Change in MCS scores**	1.4 (11.5) -37.5,42.0
	**Frequency (%)**
**Change in Marital Status**	
Newly Married	3 (2.2)
Unchanged	121 (96.3)
No Longer Married	2 (1.5)
**Change in Employment Status**	
Improved	4 (3.1)
Same	116 (88.6)
Worse	11 (8.4)
**Housing stability (moves in past year)**	
None	92 (68.2)
1	30 (22.2)
2 or more	13 (8.9)

**Table 5:** Linear regression predicting SF-12 scores at Visit 2: longitudinal cohort study (N = 131)

	**SF-12 PCS V2**		**SF-12 MCS V2**	
	***β (CI)***	***P***	***β (CI)***	***P***
SF-12 MCS V1	0.09 (−0.12-0.29)	0.416	**0.33 (0.12-0.54)**	**0.002**
SF-12 PCS V1	**0.51 (0.19-0.84)**	**0.002**	−0.25 (−0.57-0.07)	0.129
Extent Score	**0.57 (0.31-0.82)**	**0.000**	**0.64 (0.39-0.90)**	**0.000**
SF-12 MCS V1	0.15 (−0.29-0.34)	0.096	**0.48 (0.28-0.67)**	**0.000**
SF-12 PCS V1	**0.64 (0.31-0.96)**	**0.000**	−0.15 (−0.49-0.20)	0.401
Perceived Score	**0.63 (0.35-0.91)**	**0.000**	**0.53 (0.24-0.83)**	**0.000**
SF-12 MCS V1	0.18 (−0.02-0.39)	0.078	**0.50 (0.28-0.72)**	**0.000**
SF-12 PCS V1	**0.57 (0.24-0.91)**	**0.001**	−0.20 (−0.55-0.15)	0.257
Satisfaction Score	**0.44 (0.16-0.71)**	**0.002**	**0.37 (0.09-0.66)**	**0.011**

### CRIS-CAT administration study

Characteristics of the sample who participated in the Administration Study are shown in Table 3. Scores of the CRIS-CAT scales and the number of items needed to complete them are shown in Table 6. On average, the Extent subscale required more items than the Perceived Limitations and Satisfaction subscales. ICCs of each of the scales (95%CI) were 0.947 (0.908-0.969) for Extent, 0.912 (0.850-0.949) for Perceived and 0.967 (0.941-0.981) for Satisfaction. MDC values for 90 and 95% confidence are also provided in Table 6.

**Table 6:** Administration Study: Summary of raw scores, number of items used, ICCs and MDC value

	**Visit 1**	**Visit 2**			
	**Mean (SD) Range**	**Mean (SD) Range**	**ICC (CI)**	**MDC 90**	**MDC 95**
**CRIS-CAT**					
**Extent of Participation**	46.6 (10.9) 26-83	47.7 (11.2) 26-78	947 (0.908-0.969)	5.9	7.0
**Perceived Limitations**	47.0 (8.9) 35-73	47.5 (8.8) 34-77	0.912 (0.8500.949)	6.2	7.3
**Satisfaction with Participation**	46.1 (8.5) 33-78	46.3 (9.0) 35-75	0.967 (0.941-0.981)	3.6	4.3
**CRIS-CAT # of Items**					
**Extent of Participation**	14.6 (3.8) 10-20	14.7 (3.9) 10-20			
**Perceived Limitations**	10.9 (2.7) 10-20	10.7 (2.2) 10-20			
**Satisfaction with Participation**	10.4 (1.7) 10-20	10.7 (2.1) 10-20			

## Discussion

### Reliability and respondent burden

Our analyses support the reliability and validity of the CRIS-CAT. The level of scale score reliability across each CRIS-CAT scale continuum was acceptable or better for most of the scale range, but decreased at both ends of the continuum. Our results will be useful for interpreting change at the individual level. Our calculations of the MDC for each scale, are useful measures for clinicians and researchers to know because, from a statistical perspective, a patient is considered to have changed only when the difference between the previous score and the current score exceeds the MDC associated with the measurements [33]. This information is helpful in interpreting changes in scores with repeat administration.

Our study of CAT administration demonstrated that the three scales have excellent test-retest reliability and were administered with reduced respondent burden as compared to the full item set and the CRIS fixed form measure [16]. Together these findings suggest that the CRIS-CAT is feasible for adoption into research and clinical practice. Further studies of implementation of the CRIS-CAT in the clinical setting are needed.

In earlier work we found that the Extent scale had the greatest breadth of items across the entire score continuum compared with the Limitations and Satisfaction scales [16]. The Satisfaction scale showed the poorest match between the distribution of items in the item pool with the score distribution from the study sample. This pattern of item distribution corresponds to the pattern of drop off in reliability at the ends of the continuum for the Satisfaction and Limitations scale but not so much for the Extent scale. These findings suggest that the Satisfaction and Limitations scale may be less responsive to change. Further research needs to be conducted to examine responsiveness of the scales, and to identify appropriate items to add to the Satisfaction scale to reduce the ceiling effect that is likely to occur. Addition of items at the upper range of difficulty is also a potential area of improvement for the Limitations scale.

#### Concurrent and known group validity

Our concurrent and known groups’ validity results support validity of all three scales. The CRIS-CAT’s predictive validity was strong as well, as evidenced by the ability to predict 1 year ER use, quality of life (change in SF-12 scores) and diagnoses of new mental health conditions. These data suggest that assessment of community integration (as measured by the CRIS) may be clinically useful to identify Veterans at risk for adverse health events and mental health problems.

We found that the CRIS-CAT scales were most strongly correlated with QOL (R = 0.69-0.71) and with role emotional and social functioning as measured by the SF-36 (R = 0.58-0.66) In the current study we observed small correlations with both the CHART occupation scale (R = 0.27-0.32), and the CHART social integration scale (R = 0.28-0.36), suggesting that these scales measure overlapping, but different constructs than do the CRIS-CAT measures. In two earlier studies using the CRIS fixed form measure we found weak correlations between the CHART occupation scale, R = 0.23-0.25 in one study [14] and no correlation in the other study [13]. We also found weak correlations of the CHART social integration scale R = 0.17-0.26 in one study, [13] but no correlations in the other [14].

The subscales of the CRIS were designed to assess three aspects of community reintegration: frequency of participation, perceived limitations and satisfaction with participation. While these aspects are clearly related, in our view they are assessing subtly different dimensions. Our validity analyses show similar patterns of relationships for each of the three scales; suggesting that all would be appropriate for future use. Future research is needed to understand the differences between the scales and the relative merits of using them singly or in combination.

#### Predictive validity

In the current study, we did not find a relationship between CRIS-CAT scores and 1-year change in marital status, housing, or employment, as we had hypothesized. However, these findings might be attributable to the very small number of Veterans who had a change in marital status, the relatively short time frame of our follow-up and small numbers of Veterans who had changes in these domains during the follow-up period. It is possible that our findings would have been different if we had a larger sample. Future research is needed to examine the ability of the CRIS-CAT to predict changes in marital status, housing and employment.

#### Limitations

We do not believe that our findings are generalizable to all OEF/OIF Veterans because our sample was a convenience sample that was not be representative of all OEF/OIF Veterans. Only 2 people (1.5%) in our cohort became divorced during the year of the study. This figure is low, given prior reports. In 2008, for example, Cotton reported that 8.5% of married OEF/OIF women and 2.9% of men got divorced a rate that was higher than reported rates of 5.7% and 2.2% for women and men in 2000 [34]. Eleven persons (8.4%) in our cohort had worse employment at one year, while 4 persons (3.0%) had better employment. We are unaware of any national estimates of rates for employment change for OEF/OIF Veterans.

The generalizability of our findings on predictive validity is further limited because subjects lost to follow up (in the cohort study) were younger, more recently deployed, more likely to be unemployed, have lower incomes, and be unmarried. They were less likely to have children, depression, alcohol/drug abuse or new mental health diagnosis. These differences could potentially diminish the generalizability of our findings to those OEF/OIF Veterans who are younger and more recently returned from deployment.

To assess the impact of loss to follow-up on our analysis of predictive validity we conducted a sensitivity analysis to examine the impact of including cases lost to follow-up by imputing missing outcomes data. We did this by modeling PCS and separately MCS scores using all the covariates in Table 3 as well as the Visit 1 CRIS-CAT (extent, perceived, satisfaction), PCS and MCS scores using STATA 11.1’s mi commands to multiply impute 10 values for each of the SF-12 scores, based on regressions of the covariates and examined the combined results with the mi estimate command. (http://www.stata.com/stata11/mi.html).

These analyses showed that the statistically significant results of the linear regressions predicting PCS were similar for models with and without the imputed outcomes data. The statistically significant results of the linear regressions predicting MCS were similar for the CRIS-CAT Extent and Perceived Scale. However, the relationship between the CRIS-CAT satisfaction score and the MCS was no longer statistically significant (P = 0.077) in the model with the imputed MCS scores. Given these largely similar results, we believe that the bias introduced by loss to follow-up was small. However, further studies are needed to confirm our findings related to change in marital status, employment status, and housing stability.

Another limitation of our study is that we utilized real data simulations to calculate CRIS-CAT scores. Data simulations assume that respondents would have answered the subset of items selected using CAT in an identical manner to the way that they answered the same items embedded in the larger item site. Though they are considered good approximations, data simulations like these are not perfect simulations of actual CAT administration, and may overestimate these correlations [35]. Additional research is needed to examine the validity of the CRIS-CAT administered using CAT software.

## Conclusion

The CRIS-CAT demonstrated sound measurement properties including reliability, construct, known group and predictive validity. These findings support the use of this measure in assessing community reintegration. Further research is needed to examine the responsiveness of the measure, examine potential floor and ceiling effects and to identify new items to expand the number of available items at the upper end of difficulty in the Satisfaction and Perceived Limitations scales.

## Competing interests

The authors declare that they have no competing interests.

## Authors’ contributions

LR obtained funding for this study, conceptualized the design, oversaw the project, oversaw the analyses, and took the lead in writing the manuscript. MB participated in the data cleaning, data analyses, interpretation of results, writing and review of the manuscript. PN performed the IRT analyses, and analyses of reliability, created figures, and participated in interpretation of results. PP participated in conceptualization of the study design, categorization of the mental health variables, analyses of mental health outcomes, and interpretation of findings. AJ participated in conceptualization of the study design, oversaw the IRT analyses, and participated in interpretation of study results. All authors read and approved the final manuscript.

## Authors’ information

Linda Resnik, PT, PhD is a Research Health Scientist at the Providence VA Medical Center and Associate Professor (Research) in the Department of Health Services, Policy and Practice, Brown University, Providence, RI.

Matthew Borgia, MS was a graduate student in the Department of Biostatistics, Brown University at the time that this study was conducted.

## Pre-publication history

The pre-publication history for this paper can be accessed here:

http://www.biomedcentral.com/1471-2288/12/145/prepub

## Supplementary Material

Additional file 1 **Appendix A.** CRIS-CAT Item Set. Click here for file
