# Supplementary material for: Reliability, validity and administrative burden of the community reintegration of injured service members computer adaptive test (CRIS-CAT)”
Source: BMC Med Res Methodol. 2012 Sep 17;12:145. doi: 10.1186/1471-2288-12-145 (PMC3528459; doi:10.1186/1471-2288-12-145)
Supplement: Additional file 1 — Appendix A. CRIS-CAT Item Set. [file 1471-2288-12-145-S1.doc]

|  | **Group A**  **(N=69)** | **Group B**  **(N=99)** | **Group C**  **(N=332)** | **Other**  **(N=17)** | **ALL**  **(N=517)** |
| --- | --- | --- | --- | --- | --- |
|  | **Mean (SD) Range** | **Mean (SD) Range** | **Mean (SD) Range** | **Mean (SD) Range** | **Mean (SD) Range** |
| **Age** | 47.7 (8.7) 25-60 | 51.1 (7.0) 31-60 | 34.5 (9.9) 19-59 | 43.8 (8.4) 34-58 | 39.7 (11.7) 19-60 |
|  | **Frequency (%)** | **Frequency (%)** | **Frequency (%)** | **Frequency (%)** | **Frequency (%)** |
| **Gender** |  |  |  |  |  |
| Male | 49 (71.0) | 83 (83.8) | 285 (85.8) | 16 (94.1) | 433 (83.8) |
| Female | 20 (29.0) | 16 (16.2) | 47 (14.2) | 1 (5.9) | 84 (16.3) |
| **Race** |  |  |  |  |  |
| White | 50 (72.5) | 70 (71.4) | 262 (79.2) | 12 (70.6) | 394 (76.5) |
| Black | 8 (11.6) | 17 (17.4) | 16 (4.8) | 3 (17.7) | 44 (8.5) |
| Other | 9 (13.0) | 3 (3.1) | 31 (9.4) | 1 (5.9) | 44 (8.5) |
| Mixed | 2 (2.9) | 8 (8.2) | 22 (6.7) | 1 (5.9) | 33 (6.4) |
| **Hispanic** | 5 (7.3) | 5 (5.1) | 32 (9.7) | 1 (5.9) | 43 (8.4) |
| **Has** **Children** | 53 (76.8) | 73 (73.7) | 179 (53.9) | 12 (70.6) | 317 (61.3) |
| **Education** |  |  |  |  |  |
| Less than High School | 1 (1.5) | 6 (6.1) | 0 (0.0) | 0 (0.0) | 7 (1.4) |
| High School | 11 (15.9) | 32 (32.3) | 73 (21.9) | 5 (29.4) | 121 (23.4) |
| GED | 1 (1.5) | 16 (16.2) | 12 (3.6) | 1 (5.9) | 30 (5.8) |
| Some college | 30 (43.5) | 34 (34.3) | 152 (45.8) | 5 (29.4) | 221 (42.8) |
| College | 16 (23.2) | 8 (8.1) | 70 (21.1) | 5 (29.4) | 99 (19.2) |
| Post Grad | 10 (14.5) | 3 (3.0) | 25 (7.5) | 1 (5.9) | 39 (7.5) |
| **Employment status** |  |  |  |  |  |
| Unemployed | 0 (0.0) | 29 (29.3) | 67 (20.2) | 3 (17.7) | 99 (19.2) |
| Not working due to disability/medical hold | 0 (0.0) | 61 (61.6) | 23 (7.0) | 3 (17.7) | 87 (16.9) |
| Working part-time/training | 9 (13.0) | 8 (8.1) | 38 (11.5) | 1 (5.9) | 56 (10.9) |
| Working full-time | 59 (85.5) | 1 (1.0) | 200 (60.6) | 10 (58.8) | 270 (52.4) |
| Retired | 1 (1.5) | 0 (0.0) | 2 (0.6) | 0 (0.0) | 3 (0.6) |
| **Income** |  |  |  |  |  |
| Less than 25K | 10 (14.5) | 75 (75.8) | 86 (26.1) | 2 (11.8) | 173 (33.7) |
| 25k to 50k | 20 (29.0) | 17 (17.2) | 101 (30.7) | 8 (47.1) | 146 (28.4) |
| Over 50k | 39 (56.5) | 7 (7.1) | 142 (43.2) | 7 (41.2) | 195 (37.9) |
| **Marital Status** |  |  |  |  |  |
| Unmarried | 19 (27.5) | 26 (26.3) | 130 (39.2) | 6 (35.3) | 181 (35.0) |
| Married | 37 (53.6) | 17 (17.2) | 151 (45.5) | 7 (41.2) | 212 (41.0) |
| Divorced, Separated or Widowed | 13 (18.9) | 56 (56.5) | 51 (15.3) | 4 (23.5) | 124 (24.0) |
| **Residence** |  |  |  |  |  |
| Outside | 0 (0.0) | 2 (2.0) | 0 (0.0) | 0 (0.0) | 2 (0.4) |
| Staying with friend | 0 (0.0) | 11 (11.1) | 35 (10.5) | 1 (5.9) | 47 (9.1) |
| Vet Home | 0 (0.0) | 28 (28.3) | 3 (0.9) | 0 (0.0) | 31 (6.0) |
| House | 19 (27.5) | 31 (31.3) | 88 (26.5) | 6 (35.3) | 144 (27.9) |
| Apartment | 50 (72.5) | 22 (22.2) | 190 (57.2) | 10 (58.8) | 272 (52.6) |
| Other | 0 (0.0) | 5 (5.1) | 16 (4.8) | 0 (0.0) | 21 (4.1) |
| **Depression Diagnosis** | 0 (0.0) | 70 (70.7) | 83 (25.7) | 1 (5.9) | 154 (30.3) |
| **PTSD Diagnosis** | 0 (0.0) | 51 (53.1) | 90 (27.7) | 3 (17.7) | 144 (28.4) |
| **Mental Illness Diagnosis** | 1 (1.5) | 48 (50.0) | 56 (17.0) | 0 (0.0) | 105 (20.6) |
| **Alcohol/Drug abuse Diagnosis** | 0 (0.0) | 68 (68.7) | 67 (20.2) | 6 (35.3) | 141 (27.3) |

**Table 1. Descriptive characteristics of subjects in the field study by group**

|  | **Candidates for Cohort Study** | | | **Administration**  **Study** |
| --- | --- | --- | --- | --- |
|  | **Lost to follow-up (N=73)** | **Followed at 1 year**  **(N=135)** | **P** |  |
|  | **Mean (SD) Range** | **Mean (SD) Range** |  | **Mean (SD) Range** |
| **Age** | 30.6 (8.8) 20-55 | 36.7 (10.0) 21-59 | **0.000** | 48.2 (9.8) 24-59 |
| **Month since return from deployment** | 22.1 (19.9) 0-68 | 28.5 (19.0) 1-96 | **0.015** |  |
| **SF12 MCS** | 45.1 (9.2) 43.0-47.3 | 43.7 (11.9) 41.6-45.7 | 0.1863 |  |
| SF12 PCS | 41.7 (5.2) 40.4-42.9 | 41.2 (6.0) 40.1-42.2 | 0.2727 |  |
| CRISCAT Extent | 49.7 (10.1) 47.3-52.0 | 47.6 (9.0) 46.0-49.1 | 0.0638 |  |
| CRISCAT Perceived | 50.1 (9.7) 47.8-52.4 | 48.9 (7.6) 47.6-50.2 | 0.1650 |  |
| CRISCAT Satisfaction | 49.7 (9.3) 47.5-51.9 | 49.1 (8.4) 47.7-50.6 | 0.3361 |  |
|  | **Frequency (%)** | **Frequency (%)** |  | **Frequency (%)** |
| **Gender** |  |  | *0.196* |  |
| Male | 57 (78.1) | 115 (85.2) |  | 35 (70.0) |
| Female | 16 (21.9) | 20 (14.8) |  | 15 (30.0) |
| **Race** |  |  | 0.266 |  |
| White | 52 (71.2) | 111 (82.2) |  | 34 (69.4) |
| Black | 4 (5.5) | 6 (4.4) |  | 3 (6.1) |
| Other | 11 (15.1) | 10 (7.4) |  | 3 (6.1) |
| Mixed | 6 (8.2) | 8 (5.9) |  | 9 (18.4) |
| **Ethnicity** | 12 (16.4) | 11 (8.2) | 0.072 | 3 (6.1) |
| **Has** **Children** | 33 (45.2) | 82 (60.7) | **0.031** | 50 (100.0) |
| **Education** |  |  | 0.441 |  |
| Less than High School | 0 (0.0) | 0 (0.0) |  | 1 (4.0) |
| High School | 21 (28.8) | 25 (18.5) |  | 8 (16.0) |
| GED | 3 (4.1) | 6 (4.4) |  | 1 (2.0) |
| Some college | 34 (46.6) | 64 (47.4) |  | 23 (46.0) |
| College | 11 (15.1) | 29 (21.5) |  | 12 (24.0) |
| Post Grad | 4 (5.5) | 11 (8.2) |  | 4 (8.0) |
| **Employment status** |  |  | 0.058 |  |
| Unemployed | 21 (28.8) | 20 (14.8) |  | 5 (10.0) |
| Not working due to disability/medical hold | 3 (4.1) | 14 (10.4) |  | 15 (30.0) |
| Working part-time/training | 10 (13.7) | 18 (13.3) |  | 5 (10.0) |
| Working full-time | 39 (53.4) | 83 (61.5) |  | 19 (38.0) |
| Retired | 0 (0.0) | 0 (0.0) |  | 6 (12.0) |
| **Income** |  |  | **0.013** |  |
| Less than 25K | 26 (35.6) | 27 (20.0) |  | 16 (32.0) |
| 25k to 50k | 25 (34.3) | 42 (31.1) |  |  |
| Over 50k | 22 (30.1) | 66 (48.9) |  | 21 (42.0) |
| **Marital Status** |  |  | **0.014** |  |
| Unmarried | 37 (50.7) | 44 (32.6) |  | 10 (20.0) |
| Married | 22 (30.2) | 68 (50.4) |  | 25 (50.0) |
| Divorced, Separated or Widowed | 14 (19.2) | 23 (17.1) |  | 15 (30.0) |
| **Residence** |  |  | **0.002** |  |
| Outside | 0 (0.0) | 0 (0.0) |  | 0 (0.0) |
| Staying with friend | 12 (16.4) | 8 (5.9) |  | 1 (2.0) |
| Vet Home | 0 (0.0) | 2 (1.5) |  | 2 (4.0) |
| House | 18 (24.7) | 37 (27.4) |  | 17 (34.0) |
| Apartment | 34 (46.6) | 85 (63.0) |  | 29 (58.0) |
| Other | 9 (12.3) | 3 (2.2) |  | 1 (2.0) |
| **Depression Diagnosis** | 14 (19.7) | 45 (33.8) | **0.034** | 20 (40.8) |
| **PTSD Diagnosis** | 16 (22.5) | 44 (33.6) | 0.101 | 20 (40.0) |
| **Mental Illness Diagnosis** | 8 (11.3) | 23 (17.0) | 0.271 | 13 (26.0) |
| **Alcohol/Drug abuse Diagnosis** | 8 (11.1) | 32 (23.7) | **0.029** | 17 (34.0) |
| **New Diagnosis of Mental Illness** | 0 (0.0) | 35 (28.7) | **0.000** | N/A |
| **Imputed MCS score at Visit 2** | 45.0 | 44.8 |  |  |
| **Imputed PCS score at Visit 2** | 50.4 | 46.3 |  |  |

**Table 2: Characteristics of participants in the longitudinal cohort study and administration study s**:

P-values below 0.05 (*), 0.01 (**) and 0.001 (***)

| **Measure** | **Extent of Participation** | **Perceived Limitations** | **Participation Satisfaction** |
| --- | --- | --- | --- |
|  | ***R*** | ***R*** | ***R*** |
| Quality of Life Scale | 0.71 | 0.69 | 0.76 |
| Activities of Daily Living | -0.38 | -0.36 | -0.34 |
| Occupation (CHART) | 0.32 | 0.31 | 0.27 |
| Social Integration (CHART) | 0.34 | 0.28 | 0.36 |
| Physical Function (SF-36) | 0.49 | 0.38 | 0.37 |
| Role Physical (SF-36) | 0.48 | 0.45 | 0.44 |
| Role Emotional (SF-36) | 0.63 | 0.60 | 0.55 |
| Social Functional (SF-36) | 0.66 | 0.66 | 0.58 |

**Table 3: Concurrent and Discriminant Validity of CRIS-CAT scales: Pearson Product Correlations data from the field study N=500 (all p values <0.0001)**

|  | **One year**  **(N=135)** |
| --- | --- |
|  | **Mean (SD) Range** |
| **Change in PCS scores** | 5.1 (12.3) -26.3, 30.6 |
| **Change in MCS scores** | 1.4 (11.5) -37.5,42.0 |
|  | **Frequency (%)** |
| **Change in Marital Status** |  |
| Newly Married | 3 (2.2) |
| Unchanged | 121 (96.3) |
| No Longer Married | 2 (1.5) |
| **Change in Employment Status** |  |
| Improved | 4 (3.1) |
| Same | 116 (88.6) |
| Worse | 11 (8.4) |
| **Housing stability (moves in past year)** |  |
| None | 92 (68.2) |
| 1 | 30 (22.2) |
| 2 or more | 13 (8.9) |

**Table 4. Key Outcomes at Visit 2: Longitudinal cohort study**

|  | SF12 PCS V2 | | SF 12 MCS V2 | |
| --- | --- | --- | --- | --- |
|  | *β (CI)* | *P* | *β (CI)* | *P* |
| SF12 MCS V1 | 0.09  (-0.12-0.29) | 0.416 | **0.33**  **(0.12-0.54)** | **0.002** |
| SF12 PCS V1 | **0.51**  **(0.19-0.84)** | **0.002** | -0.25  (-0.57-0.07) | 0.129 |
| Extent Score | **0.57**  **(0.31-0.82)** | **0.000** | **0.64**  **(0.39-0.90)** | **0.00** |
|  |  |  |  |  |
| SF12 MCS V1 | 0.15  (-0.29-0.34) | 0.096 | **0.48**  **(0.28-0.67)** | **0.00** |
| SF12 PCS V1 | **0.64**  **(0.31-0.96)** | **0.000** | -0.15  (-0.49-0.20) | 0.401 |
| Perceived Score | **0.63**  **(0.35-0.91)** | **0.000** | **0.53**  **(0.24-0.83)** | **0.00** |
|  |  |  |  |  |
| SF12 MCS V1 | 0.18  (-0.02-0.39) | 0.078 | **0.50**  **(0.28-0.72)** | **0.000** |
| SF12 PCS V1 | **0.57**  **(0.24-0.91)** | **0.001** | -0.20  (-0.55-0.15) | 0.257 |
| Satisfaction Score | **0.44**  **(0.16-0.71)** | **0.002** | **0.37**  **(0.09-0.66)** | **0.011** |
|  |  |  |  |  |

**Table 5 Linear Regression Predicting SF-12 scores at Visit 2: Longitudinal cohort study (N=131)**

|  | **Visit 1** | **Visit 2** |  |  |  |
| --- | --- | --- | --- | --- | --- |
|  | **Mean (SD) Range** | **Mean (SD) Range** | **ICC (CI)** | **MDC 90** | **MDC 95** |
| **CRIS-CAT** |  |  |  |  |  |
| **Extent of Participation** | 46.6 (10.9) 26-83 | 47.7 (11.2) 26-78 | 947 (0.908-0.969) | 5.9 | 7.0 |
| **Perceived Limitations** | 47.0 (8.9) 35-73 | 47.5 (8.8) 34-77 | 0.912 (0.8500.949) | 6.2 | 7.3 |
| **Satisfaction with Participation** | 46.1 (8.5) 33-78 | 46.3 (9.0) 35-75 | 0.967 (0.941-0.981) | 3.6 | 4.3 |
| **CRIS-CAT # of Items** |  |  |  |  |  |
| **Extent of Participation** | 14.6 (3.8) 10-20 | 14.7 (3.9) 10-20 |  |  |  |
| **Perceived Limitations** | 10.9 (2.7) 10-20 | 10.7 (2.2) 10-20 |  |  |  |
| **Satisfaction with Participation** | 10.4 (1.7) 10-20 | 10.7 (2.1) 10-20 |  |  |  |

**Table 6: Administration Study: Summary of raw scores, number of items used, ICCs and MDC value**
